# Optical methods for cuffless blood pressure measurements

**DOI:** 10.1117/1.BIOS.3.1.010901

**Published:** 2025-10-31

**Authors:** Ariane Garrett, Ana Perez, Rivka Ayalon, John Forman, Naomi Hamburg, David Boas, Darren Roblyer

**Affiliations:** a Boston University, Department of Biomedical Engineering, Boston, Massachusetts, United States; b Boston Medical Center, Division of Nephrology, Boston, Massachusetts, United States; c Boston University School of Medicine, Whitaker Cardiovascular Institute, Boston, Massachusetts, United States

**Keywords:** blood pressure, photoplethysmography, speckle contrast optical spectroscopy, speckle plethysmography

## Abstract

**Significance:**

Accurate, noninvasive, and continuous blood pressure (BP) monitoring without a cuff is a long-standing goal in biomedical engineering, having the potential to revolutionize hypertension care. Significant effort has gone toward the development of an optical cuffless BP device, primarily using the finger photoplethysmography (PPG) signal. Despite this effort, optical-based BP monitors have yet to achieve widespread adoption primarily due to accuracy concerns. Recently, new optical techniques such as multiwavelength PPG, imaging PPG, and speckle contrast optical spectroscopy/speckle plethysmography (SCOS/SPG) have shown promise toward improved performance.

**Aim:**

We aim to review methods in optical cuffless BP estimation.

**Approach:**

We first review prior literature in finger PPG, wrist PPG, multiwavelength PPG, imaging PPG, and SCOS/SPG strategies for BP estimation. We then review the current state of commercial cuffless BP devices and accuracy standards for new devices and provide an outlook for future work.

**Results:**

Most of the effort toward the development of an optical cuffless BP monitor has centered on the use of the finger PPG signal. The methods used to quantify device performance vary widely, hindering cross-device evaluation. To mitigate this issue, future works should adhere to recently published consensus standards for performance evaluation. Emerging techniques such as imaging PPG, multiwavelength PPG, and SCOS/SPG increase the depth of physiological information used for BP monitoring and may improve cuffless BP estimation going forward.

**Conclusion:**

Optical methods for cuffless BP monitoring hold enormous promise for the healthcare and consumer spaces, but further improvements in both technology and device characterization are needed to achieve widespread adoption.

Statement of Discovery This work reviews the latest advances in optical methods for cuffless blood pressure measurements. We discuss opportunities and limitations in the field, new consensus testing standards, commercial devices, and next steps for device development, performance testing, and clinical adoption.

## 1 Introduction

Hypertension [elevated blood pressure (BP)] is the leading cause of cardiovascular disease, affecting nearly half of adults in the United States and over 1 billion adults globally.^1^^,^^2^ Blood pressure measurements consist of systolic (SBP) and diastolic (DBP) components, and hypertension is identified by an SBP greater than 130 mmHg and/or a DBP greater than 80 mmHg.^3^ Hypertension poses a major clinical and economic burden to the health care system and increases the risk of multiple adverse outcomes, including stroke, coronary artery disease, heart failure, peripheral vascular disease, and kidney disease.^2^^,^^4^ It is estimated that at least half of hypertensive individuals remain undiagnosed and thus untreated, increasing their risk for future cardiovascular disease.^2^

**Fig. 1:**
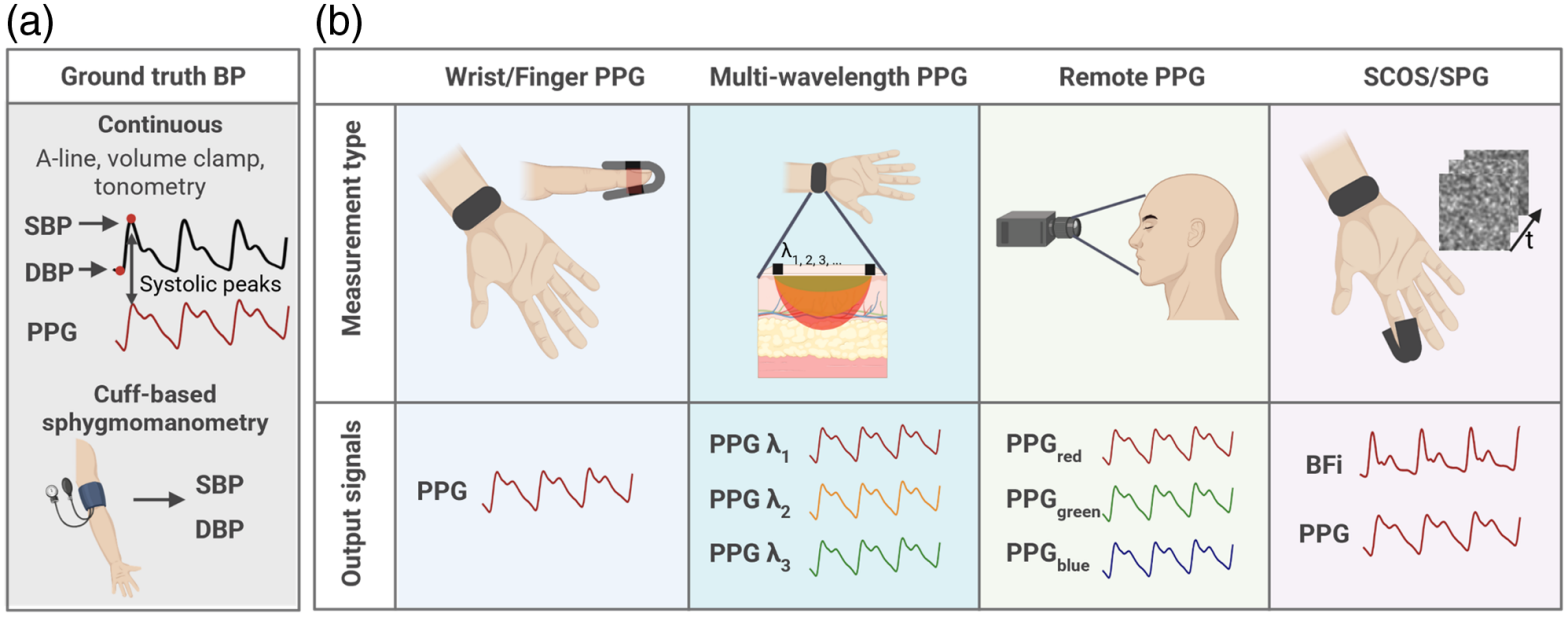
(a) Current gold-standard BP measurement techniques often used as the ground truth for optical BP estimation. (b) Overview of optical techniques for BP estimation. Most prior work has utilized the photoplethysmography (PPG) signal, including contact, noncontact (remote), and multiwavelength variations. Recently, there have been efforts to incorporate blood flood index (BFi) measurements from techniques such as speckle contrast optical spectroscopy (SCOS) and speckle plethysmography (SPG). Note that waveforms are representative and not reflective of real data. This figure was generated with BioRender.

The arterial line (A-line) is considered the gold standard for invasive measurement of blood pressure [Fig. 1(a)]. It comprises a catheter inserted into an artery (typically the radial artery), enabling real-time blood pressure monitoring via a pressure transducer connected to the catheter, as well as direct sampling of arterial blood gas measurements.^5^ Due to its invasive nature, the A-line is primarily used in critically ill patients in intensive care units (ICUs). The A-line has several associated risks, including vessel occlusion, infection, hematoma, and injury to surrounding tissues.^5^ For these reasons, although the A-line represents a highly accurate and continuous measurement of blood pressure, it is not used to diagnose hypertension in the general population.^5^

The gold standard for noninvasive BP estimation is the cuff-based sphygmomanometer, in which a cuff is placed on the upper arm over the brachial artery and inflated to measure the SBP and DBP [Fig. 1(a)]. Cuff-based BP measurements are typically taken in the clinic. Hypertension diagnosis requires an elevated BP over at least two visits. However, for patients in whom there is suspected white coat hypertension, masked hypertension, or from whom the clinician desires a more precise picture of their day-to-day BP, ambulatory blood pressure monitoring (ABPM) may be prescribed. To perform ABPM, a patient wears an automatic BP cuff for 24 or more hours that records BP measurements typically every 30 to 60 min. There is a large body of evidence indicating that frequent BP measurements outside of the clinic provide a more robust assessment of a person’s “usual” BP compared with a single measurement acquired in the clinician’s office.^6^ In addition, the difference between daytime and nighttime BP is strongly correlated with cardiovascular risk prediction, and this can only be captured with ABPM.^7^^,^^8^ However, current cuff-based ABPM measurements are loud, uncomfortable, and generally disruptive to patients’ daily lives, especially their sleep.^9^ Clinics may only have a few ABPM devices available, leading to long wait times. These factors together have limited widespread adoption of ABPM. Alternative methods for continuous cuffless BP monitoring are highly desired by the medical community, but, due to the challenge of accurately estimating BP without a cuff, no new technologies to date have seen widespread clinical adoption.^2^^,^^9^^,^^10^

Many emerging technologies for cuffless BP utilize optical measurements because they are generally unobtrusive and inexpensive and have shown promise for accurate estimation of BP under certain conditions, such as at rest or over short periods of time.^2^^,^^11^^–^^13^ Figure 1(b) shows optical measurement techniques for BP estimation. Most research in this area has centered on photoplethysmography (PPG).^10^^–^^12^ The PPG signal originates from oscillatory changes in tissue light absorption during the cardiac cycle. As the volume fraction of blood in tissue varies with each heartbeat, the measured light intensity changes in turn. This relationship can be described through the modified Beer–Lambert law, where the absorbance ($A_{t}$) of light at a given time point can be expressed as

$$A_{t}=\ln(\frac{I_{0}}{I_{t}})=\epsilon_{\lambda }C_{t}l+G_{\lambda },$$

for the simple case of a single absorbing species. $I_{0}$ is the incident light intensity (often approximated as the mean of measured light intensity over a given time period), $I_{t}$ is the intensity at a given time t, $\epsilon_{\lambda }$ is the extinction coefficient of the absorber at a specified wavelength, and $C_{t}$ is the concentration of the absorber at the specified time t. In tissue, there are multiple absorbers, including oxy and deoxy-hemoglobin, water, lipids, and others. However, nonhemoglobin absorbers are typically thought to remain constant during the cardiac cycle, contributing to the DC (steady-state) component of the PPG signal but not to modulations over shorter time periods.^13^ The parameter G, which is related to scattering losses, is also considered to remain constant during the cardiac cycle. The increase in blood volume fraction during systole, caused by the expansion of arteries and arterioles, causes the partial pathlength (l) of light through blood-containing vessels to increase, which in turn increases optical absorbance due to hemoglobin. The measured change in absorbance can be interpreted as a relative measure of blood volume change in the tissue. Because the PPG signal provides a dynamic measure of the peripheral cardiovascular system, it has long been explored for its ability to predict BP.

In this work, we will begin by reviewing efforts to use the PPG signal on the finger and wrist to estimate BP. We will then discuss emerging strategies using remote PPG, multiwavelength PPG, and speckle contrast optical spectroscopy (SCOS) (Fig. 1). This review focuses on single-site, single-mode strategies, and we note that multi-modal techniques that incorporate non-optical methods such as ECG^14^ are generally beyond the scope of this review. However, we note that several included studies do incorporate multi-site or multi-mode measurements as a secondary component. Finally, we will discuss consensus standards for testing new cuffless BP technologies and recently cleared commercial devices. We will end by reviewing the remaining challenges and opportunities for optical BP monitoring.

## 2 Optical Methods for BP Estimation

There are hundreds of studies that explore optical methods for BP estimation spanning several decades. In this review, we focus on a subset of prior works that represent both important and recent advancements to the field. Key information about these papers is shown in Table 1 (a more detailed version of this table is included as Table S1 in the Supplementary Material). This includes the type of measurement, the number and type of subjects included, the BP prediction errors, the calibration procedure, and whether there is data leakage. Here, calibration refers to a gold standard BP measurement that was used to tune a BP estimation model at or near the testing measurement period, which was separate from a preceding training measurement period. Data leakage refers to a scenario in which data from the so-called “same measurement” and subject appear in both the testing and training sets. We define the “same measurement” as any measurement occurring within the same 24-h window. As we will discuss later in this review, data leakage can greatly reduce BP estimation errors but may not be reflective of the model/device performance in a real-world scenario.^27^^,^^35^ Across the papers reviewed, multiple methods are used to assess model performance, including correlation coefficient, mean absolute error, mean error, and standard deviation. In addition, papers often use terms such as “accuracy” and “error” to describe their results. In this work, “accuracy” is used to describe how well the predicted BP values match the ground truth BP. “Error” is used to describe the difference between the predicted and true BP, in whatever method the authors choose to report those errors. A single method of reporting errors is not superior to others; however, the correlation coefficient should not be the sole reported error metric as it is possible that the predicted and true BP can be well correlated but still have large errors. In addition, the mean error should not be reported without the standard deviation as the mean error may be small if large positive and negative errors cancel each other out. Specific published guidelines for reporting errors are discussed later in this review.

**Table 1:** Optical BP monitoring studies discussed in this review. A more detailed version of this table is included in the Supplementary Material.

Ref.	Site	Optical measurement	Analysis method	# of subjects	Calibration^a^	Data leakage^b^	Errors^c^ (mmHg)
Choudhury et al.^15^	Finger	PPG	PM	32	N	N	S: 0.78 ± 13.1
							D: 0.59 ± 10.23
Xing et al.^16^	Finger	PPG	F-ML	1249	N	N	$S_{y}$^d^: 2.1 ± 13.6
							$D_{y}$: 2.3 ± 9.5
							$S_{o}$: 5.5 ± 15.5
							$D_{o}$: 2.6 ± 9.3
Kurylyak et al.^17^	Finger	PPG	F-DL	>15,000 samples	N	NS	S: 3.8 ± 3.46
							D: 2.21 ± 2.09
Mousavi et al.^18^	Finger	PPG	WS-ML	441	N	NS	S: 0.187 ± 4.1
							D: −0.050 ± 8.9
Slapnicar et al.^19^	Finger	PPG	WS-DL	510	Y	N	S: 9.43
							D: 6.88
Paliakaite et al.^20^	Finger + Wrist	PPG	F-ML	22	N	Y	$S_{f}$^e^: 0.47 ± 10.44
							$S_{w}$: 1.0 ± 12.86
Yao et al.^21^	Wrist	PPG	F-DL	33	N	Y	S: −0.07 ±4.47
							D: 0.00 ± 3.61
Wang et al.^22^	Wrist	PPG	F-DL	18	N	N	S: 0.44 ± 6
							D: −0.5 ± 6.2
Goudarzi et al.^23^	Face	iPPG	F-ML	40	N	NS	S: 0.45 ± 12.39
							D: −0.2 ± 6.41
Rong and Li^24^	Face	iPPG	F-ML	189	N	NS	S: 2.1 ± 3.35
							D: 0.79 ± 2.58
Wu et al.^25^	Face	iPPG	F-DL	1143	N	N	S: 11.54 ± 1.32
							D: 8.09 ± 0.7
Huang et al.^26^	Face + Palm	iPPG (transfer learning from MIMIC II)	F-DL	13	N	NS	S: 14.02
							D: 7.38
Schrumpf et al.^27^	Face	iPPG (transfer learning from MIMIC III)	WS-DL	50	Y	N	S: 12.7
							D: 10.5
Liu et al.^28^	Finger	MWPPG	PM	20	N	Y	S: 2.9
Liu et al.^29^	Finger	MWPPG	PM	20	N	Y	S: 2.2
							D: 1.4
Liu et al.^30^	Finger	MWPPG	PM	22	N	N	S: 1.44 ± 6.89
							D: −1.00±6.71
Cui et al.^31^	Finger	MWPPG	WS-DL	162	N	Y	S: 0.22±5.28
							D: 0.71±2.43
Chang et al.^32^	Finger	MWPPG	F-ML	10	—	—	Correlation with BP: S: r=0.79
							D: r=0.78
Garrett et al.^33^	Finger + Wrist	SCOS/SPG + PPG	F-ML	30	N	Y	S: 2.26
							D: 1.69
							S: $9.85_{L}$^f^
							D: $7.68_{L}$
Ellington et al.^34^	Finger	SCOS/SPG	F-ML	8	Y	N	S: $2.8_{\text{rest}}$
							S: $2.2_{\mathrm{LE}}$^g^
							S: $19.3_{\mathrm{HE}}$

PM, physiological model; F, feature-based; WS, whole signal-based; DL, deep learning; ML, machine learning; S, systolic BP; D, diastolic BP; NS, not specified.

a Calibration refers to the use of a gold standard BP measurement to tune a BP estimation model at or near the testing measurement period, and separately from the training measurement period.

b Data leakage refers to the mixing of testing and training sets such that data from the same measurement and subject may be seen in both the testing and training sets. We define measurements to be the “same measurement” as any measurements occurring within the same 24-h window.

c Table rows display information corresponding to the measurement/technique with the lowest achieved errors—or to those that correspond to what we deem the most significant result—per reference paper. Unless marked otherwise, errors displayed as single numbers correspond to mean absolute error or mean absolute deviation (MAE or MAD), whereas errors displayed as XX ± XX are mean errors.

d o and s subscripts indicate old (>50 years) or young subjects, respectively.

e f and w subscripts indicate measurements taken at the finger or wrist, respectively.

f L subscript refers to results from the longitudinal measurement, in which subjects were remeasured after several weeks.

g LE and HE subscripts refer to light and heavy exercise, respectively.

### 2.1 Finger PPG

**Fig. 2:**
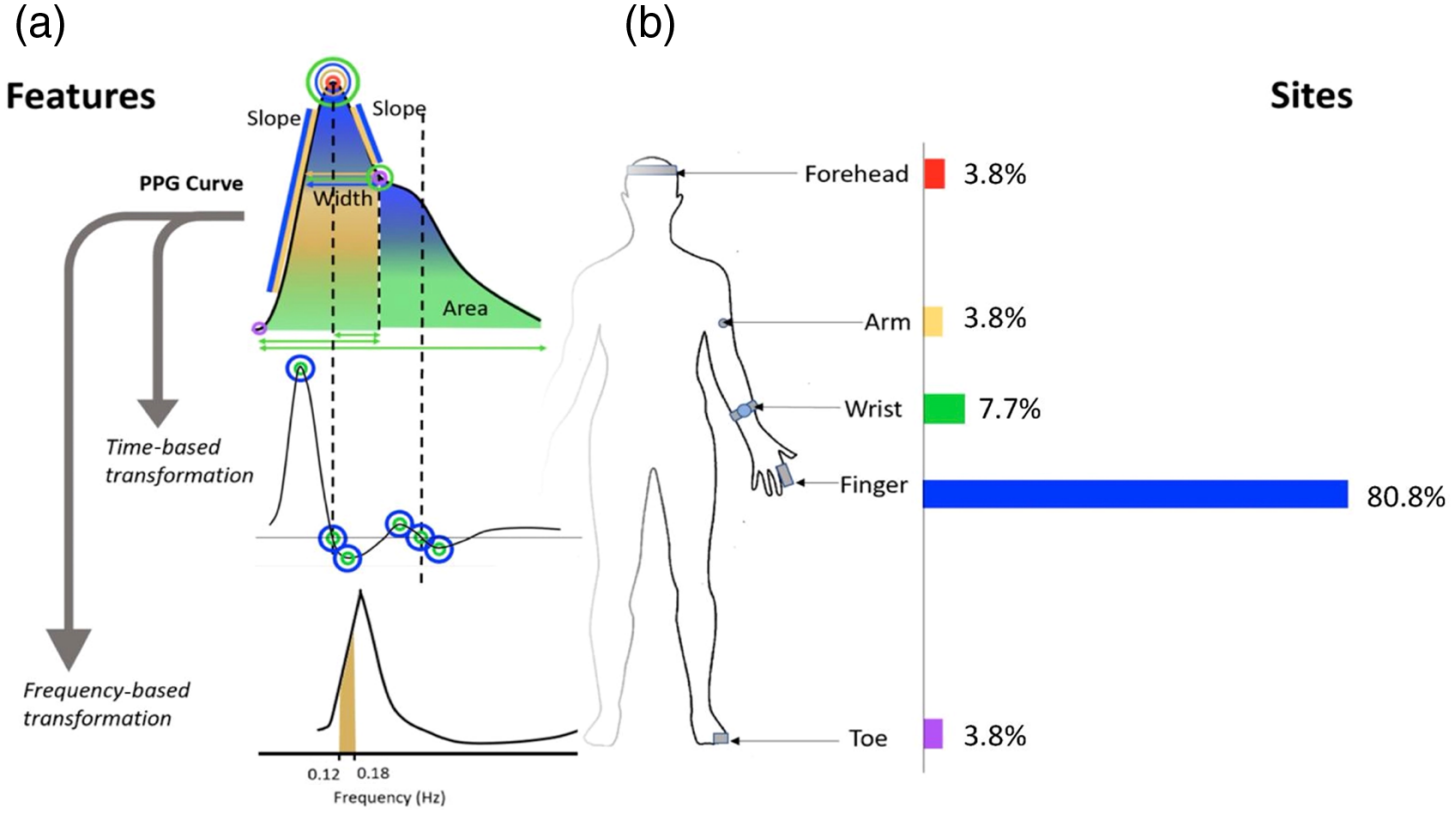
(a) PPG waveform. Features related to slope, area under the curve, and width are typically used to estimate BP. Features related to derivatives of the waveform and frequency domain features are also common. (b) A survey of papers from 2010 to 2019 by Hosanee et al. found that more than 80% of prior research estimating BP from PPG waveforms used the finger PPG signal. Adapted with permission from Ref. 36.

The PPG waveform has been analyzed for a wide range of features related to BP [Fig. 2(a)] and has been measured on a range of anatomic locations throughout the body [Fig. 2(b)]. The finger is the most common anatomical site for measurement, typically performed in transmission mode, where the source and detector are positioned on opposite sides of the finger, using near-infrared (NIR) light as the illumination source. The popularity of the finger is likely due to at least two reasons. First, this measurement site is already widely used in clinical settings for commercial pulse oximetry, which enables the use of PPG signals from large clinical databases. The most popular is the MIMIC II data base, which consists of more than 20,000 sets of continuous A-line BP, PPG, and ECG signals from patients in the ICU. Although the use of such large, pre-existing databases is convenient for developing ML models, there may be a disadvantage to using data without control of how measurements were taken. Second, the finger PPG signal tends to have a higher signal-to-noise ratio (SNR) and is less dependent on precise optode placement compared with PPG measurements on other parts of the body [Fig. 2(b)].^20^^,^^37^ There are hundreds of studies that utilize finger PPG for BP estimation, and there are already several excellent reviews that discuss these works comprehensively.^36^^,^^38^^–^^43^ Here, we will provide a brief overview of this literature with a focus on several key results.

The relationship between BP and PPG signal is nonlinear and not fully understood.^13^^,^^15^^,^^44^ Efforts to characterize this relationship have included both empirical approaches and model-based analyses. For example, the Windkessel model uses an electrical circuit analogy to describe the hemodynamic behavior of large arteries, with blood flow, peripheral resistance, and arterial compliance modeled as electrical current (I), resistance (R), and capacitance (C), respectively. Choudhury et al.^15^ adapted the Windkessel model and derived an expression for systolic and diastolic pressure dependent on I, R, and C. R and C were estimated from PPG features using linear regression. They tested their model on 32 patients in the ICU with A-line BP as the ground truth. The model achieved mean errors of 0.78±13.1  mmHg for SBP and 0.59±10.2  mmHg for DBP.^15^ However, this kind of model-based analysis is limited by simplifying assumptions that may not accurately reflect the physiological complexities of vessels and hemodynamics in the periphery.

Many recent papers employ machine learning or deep learning to predict BP from PPG, an approach that requires no explicit assumptions about the underlying physiological relationships. For example, Xing et al.^16^ explored the use of a random forest algorithm for estimating BP from 1249 subjects. They employed principal component analysis (PCA) for feature extraction, identifying four principal components from the PPG waveform and eight from the second derivative of the PPG. The study explored various demographic (age, BMI, height) and physiological subgroups and concluded that incorporating biometric features enhanced BP estimation, particularly in populations with unstable perfusion levels. Calibration improved estimation accuracy significantly. The study reported mean errors for the calibrated model of −0.1±9.5  mmHg (young) and 0.0±11.2  mmHg (old) for SBP, and −0.1±9.0  mmHg (young) and −0.2±7.2  mmHg (old)^16^ for DBP. On the other hand, uncalibrated errors were 2.1±13.6  mmHg (young) and 5.5±15.5  mmHg (old) for SBP and 2.3±9.5 (young: <50 years) and 2.6±9.3 (old: >50 years) for DBP.

Kurylyak et al. were the first to use neural networks to estimate BP from PPG. The authors utilized a feature-based artificial neural network, which was trained on the MIMIC database (version unspecified), comprising 15,000 PPG samples with corresponding BP measurements. A total of 21 features were extracted, primarily derived from the width of systolic and diastolic peaks, and served as input neurons to a multilayer feed-forward artificial neural network with two output neurons corresponding to SBP and DBP. The model achieved low mean errors of 3.8±3.46  mmHg for SBP and 2.21±2.09  mmHg for DBP.^17^

Although these prior works employed feature extraction, others have utilized the entire waveform as the input to the model. This strategy potentially avoids information loss from predefined feature selection and enables the model to learn directly from all available signal characteristics. Mousavi et al.^18^ adopted a whole-signal machine learning approach to estimate BP from PPG signals using the MIMIC database. The study included 1323 15-s PPG segments from 441 individuals. Data between consecutive systolic peaks were extracted and reduced to 43 dimensions via PCA. The study compared several regression algorithms, including decision tree, support vector, random forest, and adaptive boosting (Adaboost) regression. Adaboost achieved the best performance, with mean errors of 0.187±4.1  mmHg for SBP and −0.050±8.9  mmHg for DBP. The algorithm produced good results for both noisy and high SNR PPG signals, representing a potential advantage over feature-based techniques in which features may be difficult to extract from noisy waveforms.

Slapničar et al.^19^ also used whole PPG signals and their derivatives but explored a deep learning approach. Their study involved a dataset of 510 subjects, with both feature-based (random forest) and whole-signal (ResNet) models tested. Personalization of the models (tuning with subject-specific data) significantly improved performance, reducing errors in BP estimation. Without personalization, the ResNet model achieved mean errors of 15.41 mmHg (SBP) and 12.38 mmHg (DBP), whereas with personalization, these errors decreased to 9.43 mmHg (SBP) and 6.88 mmHg (DBP). The feature-based random forest method showed higher errors, particularly without personalization.

These studies highlighted the diverse methodologies used in noninvasive BP estimation from finger PPG signals, ranging from model-based analyses to whole-signal deep learning models. Commonly used features include the slope of the systolic upstroke, the temporal location and amplitude of the dicrotic notch, PPG signal width, the amplitude of the second derivative PPG signal, and the time interval between systolic peaks.^16^^,^^45^^–^^47^ In general, although feature-based methods provide more interpretable models, whole-signal approaches may be less prone to information loss and more robust to noise. In addition, the integration of biometric information, calibration strategies, and consideration of perfusion status have been shown to further enhance estimation accuracy. While using large databases such as MIMIC II enables the construction of large machine/deep learning models, several factors must be considered. First, the ground truth of these datasets is A-line BP, which is typically acquired from unhealthy patients in the ICU who may be under general anesthesia and therefore may not be representative of the general population. In addition, the PPG and BP instances from individual subjects are frequently not separated between the training and testing datasets, which can lead to an overestimation of model performance.^27^ This is referred to as data leakage. Schrumpf et al.^27^ compared model performance using two approaches: one where data from individual subjects from the MIMIC II dataset were split between training and test sets (“mixed”), and another where each subject’s data were kept entirely separate (“unmixed”). They found that models trained on the mixed dataset performed significantly better than those using the unmixed approach.^27^

Overall, these studies demonstrated the potential of finger PPG for noninvasive blood pressure estimation while also highlighting critical methodological considerations—such as model interpretability, data quality, and subject-wise data separation—that must be addressed to ensure robust and clinically applicable results.

### 2.2 Wrist PPG

**Fig. 3:**
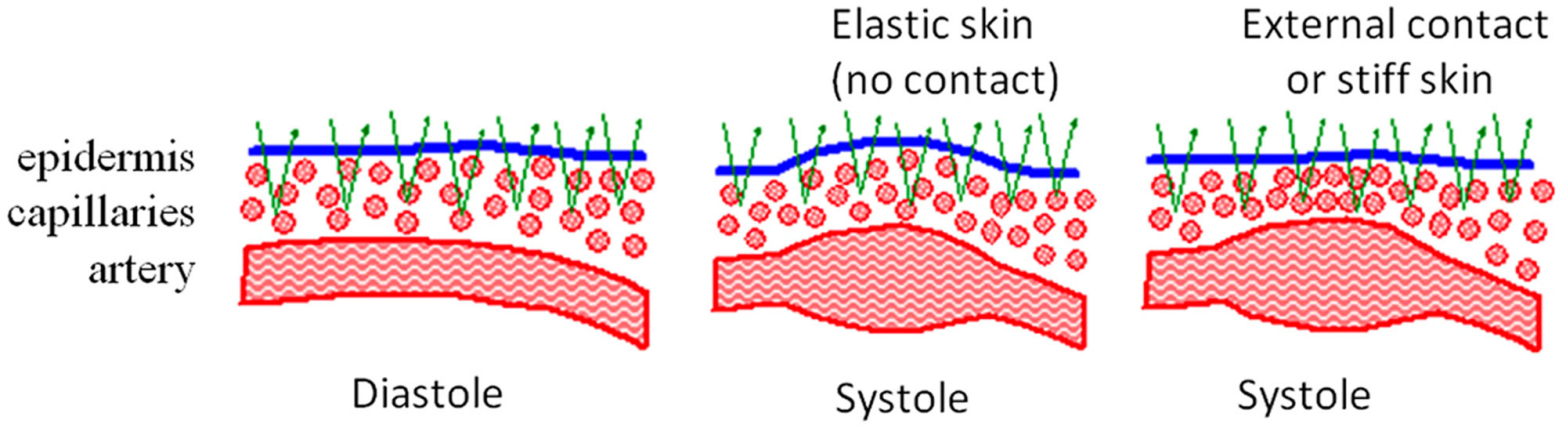
Theory for the origin of reflective PPG signal at visible wavelengths proposed by Kamshilin et al.^48^ Visible light penetration is typically limited to the epidermis and superficial capillaries. In this theory, the PPG signal may arise from the compression of the capillaries by the deeper arteries. Adapted with permission from Ref. 48.

Compared with finger PPG, there is significantly less research on estimating BP from wrist PPG signals. That said, estimating BP from the wrist is appealing because wrist-worn consumer wearables are already widely accepted and may be less obtrusive to daily activities compared with a finger wearable. Compared with finger PPG, wrist PPG is obtained in reflective mode (source and detector on the same side of the tissue) due to the thickness of the wrist (Fig. 3). This enables wrist PPG signals to be obtained from a wider range of wavelengths, ranging from visible to NIR. Green light (∼530  nm) is frequently used in these measurements, prompting a robust debate about the origins of reflectance PPG signals on the wrist.^48^^–^^50^ Due to the low penetration depth of green light into tissue (some estimates indicate a penetration depth of ∼0.6  mm),^51^^,^^52^ the light only reaches the dermis, which contains capillary loops and capillary beds, but not deeper arterioles and arteries such as the commonly targeted radial artery.^48^^,^^52^^–^^54^ Despite the attenuation of the pressure pulse waveform in capillary beds (where pulsatility is believed to be minimal), green/visible light still detects a clear reflectance PPG signal.^55^

To explain this, Kamshilin and Margaryants^48^ hypothesized that this PPG signal arises from the compression of the capillaries by deeper vasculature with each cardiac pulse (Fig. 3). As the volume of blood in the radial artery increases with each pulse, the capillary beds between the deeper vasculature and the sensor are compressed, increasing the concentration of capillaries and therefore hemoglobin in the measured tissue volume.^48^^,^^50^^,^^56^ This theory also offers an explanation for inverted, or counter-phase, PPG waveforms, which have been observed by several groups.^48^^,^^56^^–^^58^ The conventional PPG model cannot offer a simple explanation for the presence of these inverted waveforms. Kamshilin and Margaryants suggested that the inverted waveforms arise from expansion of the dermis in areas adjacent to dermal compression, similar to elastic rubber,^48^^,^^50^ where increased pressure to the center of the rubber will lead to expansion in adjacent areas. Moço et al. countered this theory, stating that the PPG signal at shorter wavelengths originates from pulsatile arterioles in the dermis, which may be sampled by green light.^59^ They performed Monte Carlo simulations and showed that, although the majority of green light only targets capillary beds and loops, some photons reach dermal arterioles, and this may provide enough pulsatile signal to explain the presence of the PPG signal at shorter wavelengths. They also offered an alternative theory for the presence of inverted waveforms and suggested that they are a result of ballistocardiogram artifacts.^60^ More research is needed to fully understand the origin of reflectance PPG signals and how this is dependent on wavelength, source detector separation, and sensor location. Here, we will review several papers using wrist PPG signals to estimate BP.

Some previous studies have shown that BP measurements derived from wrist PPG signals may be less accurate than PPG measured at the finger. For example, Paliakaitė et al.^20^ compared the use of wrist and finger PPG for BP estimation in a cohort of 22 subjects at rest and during a cold pressor challenge. They acquired reflectance PPG measurements at a green wavelength on the wrist and red/NIR transmission measurements on the finger, using cuff-based BP measurements as the ground truth. BP was estimated using linear regression models with features extracted from the waveforms as inputs. Their results showed that finger-based PPG performed better, with an SBP mean error of 0.47±10.44  mmHg for the finger-based measurements and 1.0±12.86  mmHg for wrist-based measurements. The authors suggested that different models may be necessary for wrist and finger-based BP measurements and highlighted the importance of sensor placement in optimizing BP estimation accuracy.^20^

Similar to the findings of Xing et al.,^16^ incorporating a subject’s demographic data as additional features for machine learning-based BP prediction has been shown to improve the accuracy of BP estimation from wrist-based PPG signals. Yao et al.^21^ presented a multidimensional feature fusion method for wrist PPG-based BP estimation. The study included 33 subjects, with BP measurements taken using a cuff as the ground truth. PPG signals were acquired at red and NIR wavelengths on the dorsal side of the wrist. The authors utilized subject-specific artificial neural networks (ANN) to predict BP and incorporated both optical features and demographic data such as age, height, and weight. The model achieved minimal errors, with 0.00±3.61  mmHg for DBP and −0.07±4.47  mmHg for SBP, and found that incorporating demographic data significantly enhanced the accuracy of BP predictions. In addition, they measured two subjects on days 3, 6, 8, 12, and 15 after the initial measurement to investigate the ability of the device to track BP over time. In this case, the model was trained on all subjects from the initial cohort, including the two test subjects. For the subsequent measurements, the model predicted DBP and SBP with 85% to 100% of pulses within 5 mmHg of the reference BP.^21^

There are conflicting reports about which area of the wrist offers the highest SNR for PPG measurements, and it is likely dependent on wavelength, source detector separation, and specific placement of the optodes. Several studies have suggested that measurement over the radial or ulnar arteries on the palmar side offers higher SNR.^48^^,^^50^^,^^61^ Wang et al.^22^ proposed a novel method for continuous BP monitoring using a wrist device equipped with PPG sensors on both the dorsal and palmar sides. The PPG signals were acquired at 525 nm, and measurements were taken on 18 subjects. Key features extracted from the PPG signals included the baseline difference between palmar and dorsal PPG signals, systolic and diastolic time intervals, and the ratio of the AC and DC components of the signal. They used a multilayer perceptron neural network to predict BP, evaluated through a leave-one-subject-out approach. The results showed a mean error of 0.44±6  mmHg for SBP and −0.5±6.2  mmHg for DBP.^22^

The studies reviewed demonstrate the growing potential of wrist-based PPG for continuous BP monitoring. Research has explored various sensor placements, multimodal approaches, feature extraction techniques, and machine learning models to improve BP prediction accuracy. Although wrist-based PPG sensors are promising for noninvasive, real-time BP monitoring, challenges remain in optimizing sensor placement. Research toward a more complete understanding of the origins of the wrist PPG signal may enable improved BP estimation from the wrist in the future.

### 2.3 Imaging PPG

The use of imaging photoplethysmography (iPPG) for blood pressure (BP) estimation has garnered significant attention in recent years, at least partly driven by the COVID-19 pandemic and a desire for contactless health monitoring.^25^ There are several advantages of iPPG. Measurements do not depend on adequate contact between the sensor and tissue, and a wide area of tissue can be imaged, thus making iPPG independent of sensor placement. In addition, in many configurations, only ambient light is required.^23^^,^^54^ On the other hand, iPPG signals are highly sensitive to subject motion and typically have lower SNR than contact PPG signals.^62^

As mentioned previously, visible light does not penetrate into deeper vasculature, and reflective PPG signals obtained with shorter wavelengths are potentially arising from the compression of capillary beds by pulsatile arteries, rather than directly from volume changes in the arteries themselves.^48^^,^^56^ Without the external pressure of the sensor on the tissue, the capillaries are compressed less, and thus, the SNR of the reflective PPG signal decreases (Fig. 3). Kamshilin et al.^56^ further supported this theory by obtaining iPPG measurements through a glass sheet that applied different levels of pressure to the tissue. As pressure increased, the SNR of the iPPG signal increased, supporting their hypothesis that mechanical compression of capillaries enhances the PPG signal.^56^ Alternatively, Moço et al. suggested that compression improved the SNR of the PPG signal by blanching the upper layers of tissue, thus allowing visible light to penetrate more deeply.^59^ In both cases, the authors agreed that compression improves the SNR of the PPG signal obtained at visible wavelengths. Nonetheless, it is possible to obtain an iPPG signal without external pressure, and several groups have explored the use of this signal for remote BP estimation.^23^^–^^27^ With the exception of Kamshilin et al., the papers reviewed here acquire iPPG signals from the face. To our knowledge, there has not been a comprehensive comparison of iPPG signal quality across different body locations. However, the face is typically uncovered, making it a practical site for iPPG measurements in real-world scenarios.

**Fig. 4:**
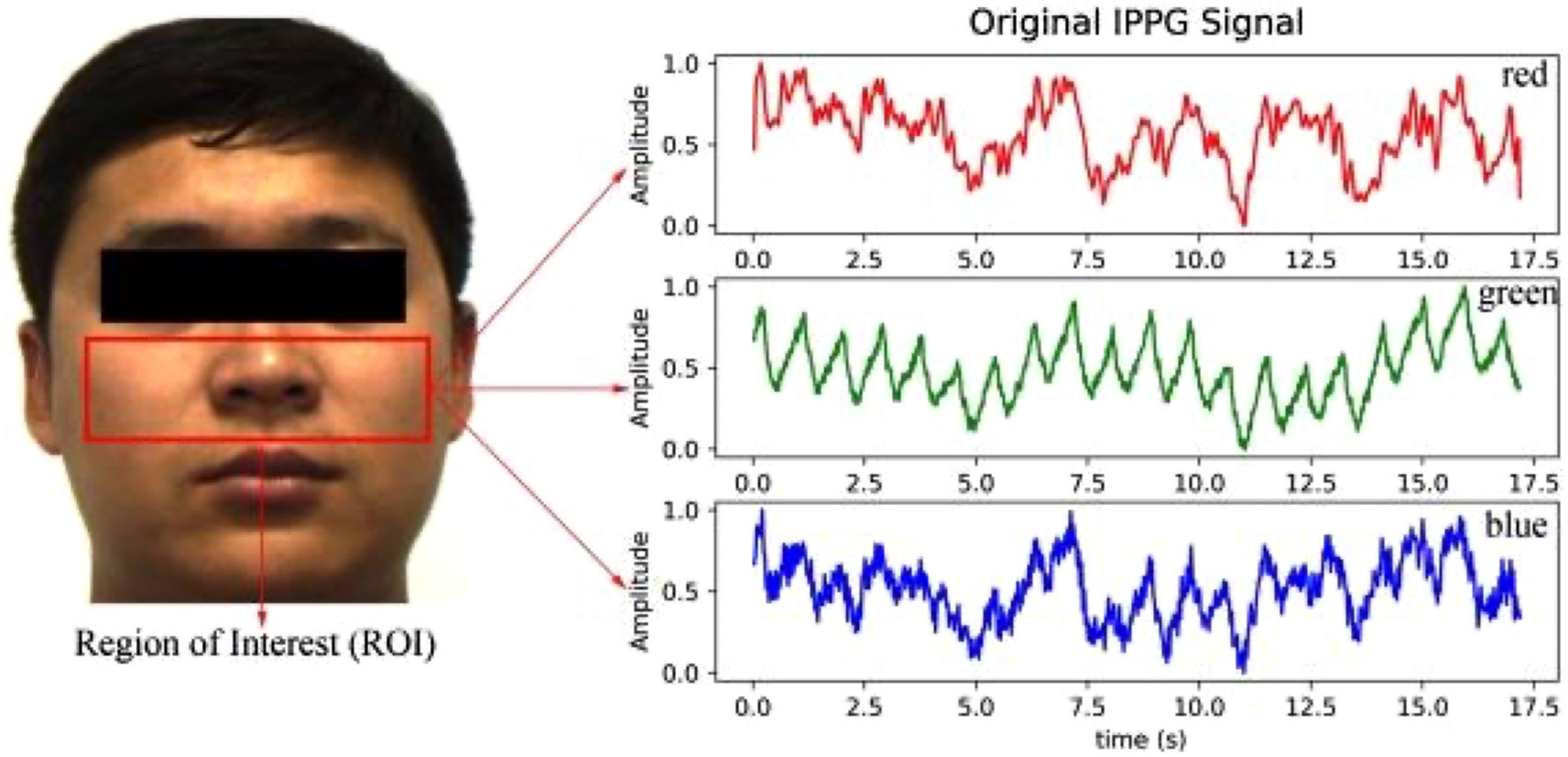
iPPG signals acquired from the cheek region with a color (RGB) camera. The green channel was shown to have a more robust signal quality compared with the red and blue channels. Adapted with permission from Ref. 24.

One challenge for iPPG using ambient light is determining how to appropriately combine and interpret the RGB channels when using a color camera. In one example, Goudarzi et al.^23^ collected iPPG signals from 40 male subjects on the forehead with ambient light as the source. They applied independent component analysis to the RGB channels of the iPPG signal to combine them into a single signal and then used random forest regression to predict BP. The results showed modest prediction performance, with SBP errors of 0.45±12.39  mmHg and DBP errors of −0.2±6.41  mmHg.^23^ In a larger study, Rong and Li^24^ measured iPPG signals from 189 volunteers using an RGB camera and used an Omron BP cuff as the ground truth. In this case, the iPPG signal was taken as the green channel of the camera (Fig. 4).^24^ They compared four machine learning models and found that support vector regression provided the best prediction results, with SBP errors of 2.1±3.35  mmHg and DBP errors of 0.79±2.58  mmHg.

The wide field of view of iPPG images also enables the extraction of pulse transit times (PTTs) from a single iPPG measurement. PTTs have previously been correlated with BP for contact-based measurements.^63^^–^^65^ Wu et al.^25^ introduced a calibration-free iPPG system that utilized PTTs calculated from the cheek to the forehead to estimate BP. Facial images were collected from a large cohort of 1143 subjects. The deep learning model incorporated features such as heart rate, heart rate variability, PTT, and body mass index derived from the image. They achieved MAE values of 11.54±1.32  mmHg for SBP and 8.09±0.7  mmHg for DBP.^25^ Huang et al.^26^ also used PTTs from iPPG, utilizing face-to-palm PTTs imaged with the subject holding their palm up next to their face. They applied transfer learning from the MIMIC II dataset to train a K-nearest neighbors model, which was then used to estimate BP using iPPG signals from 13 subjects. The system achieved MAEs of 14.02 mmHg for SBP and 7.38 mmHg for DBP. The modest performance suggests that models trained on finger PPG signals may not be generalizable to estimating BP from iPPG signals.^26^

Schrumpf et al.^27^ also investigated applying models trained on finger PPG to iPPG data. They collected iPPG signals from 50 subjects in the ICU, and trained several neural networks on the MIMIC-III dataset, which contains thousands of instances of finger PPG and A-line BP. They found that applying these models directly to iPPG data without fine tuning produced poor results, with SBP errors of 28.1 to 33.5 mmHg and DBP errors of 11.5 to 13.8 mmHg. Fine tuning their models for the iPPG data lowered the errors to 12.7 to 15.2 mmHg for SBP and 10.3 to 11.2 mmHg for DBP. This further suggests that models trained on finger PPG data cannot be directly applied to iPPG data.^27^

Together, these studies illustrated the unique challenges and advantages of BP estimation using iPPG. iPPG allows for the measurement of the PPG signal in ambient light; however, the signals are typically noisier, so more robust data processing strategies must be applied.^24^ The wide field of view of iPPG images allows for PTTs to be calculated from single images, rather than with two distinct measurement sites as is typical with contact PPG.^25^^,^^26^ Last, preliminary evidence suggests that neural networks trained on finger PPG signals cannot be used to predict BP from iPPG signals accurately. This may be due to differences in noise level, measurement location, wavelength of the source, and ultimately the tissue volume from which the signal originates.

### 2.4 Multiwavelength PPG

Although optical blood pressure estimation has long been dominated by single-wavelength PPG, there has been an increasing interest in multiwavelength photoplethysmography (MWPPG). By exploiting the wavelength-dependent depths at which light can penetrate the skin, MWPPG signals allow for the isolation of blood volume changes occurring at the capillary, arteriole, and/or arterial levels of vasculature.^28^ Furthermore, single-wavelength techniques may suffer from greater signal quality variability originating from subject and trial-dependent factors such as skin color and contact pressure. MWPPG may help alleviate this problem by providing a range of wavelengths over which the best quality signal can be chosen.^32^ In addition, measuring several wavelengths may have the added benefit of collecting other signals concurrently with PPG, such as ${\mathrm{SpO}}_{2}$.^32^ Similar to single-wavelength PPG, MWPPG may be used in either physiological or machine/deep learning models for blood pressure estimation. In this section, we will review a variety of such techniques.

**Fig. 5:**
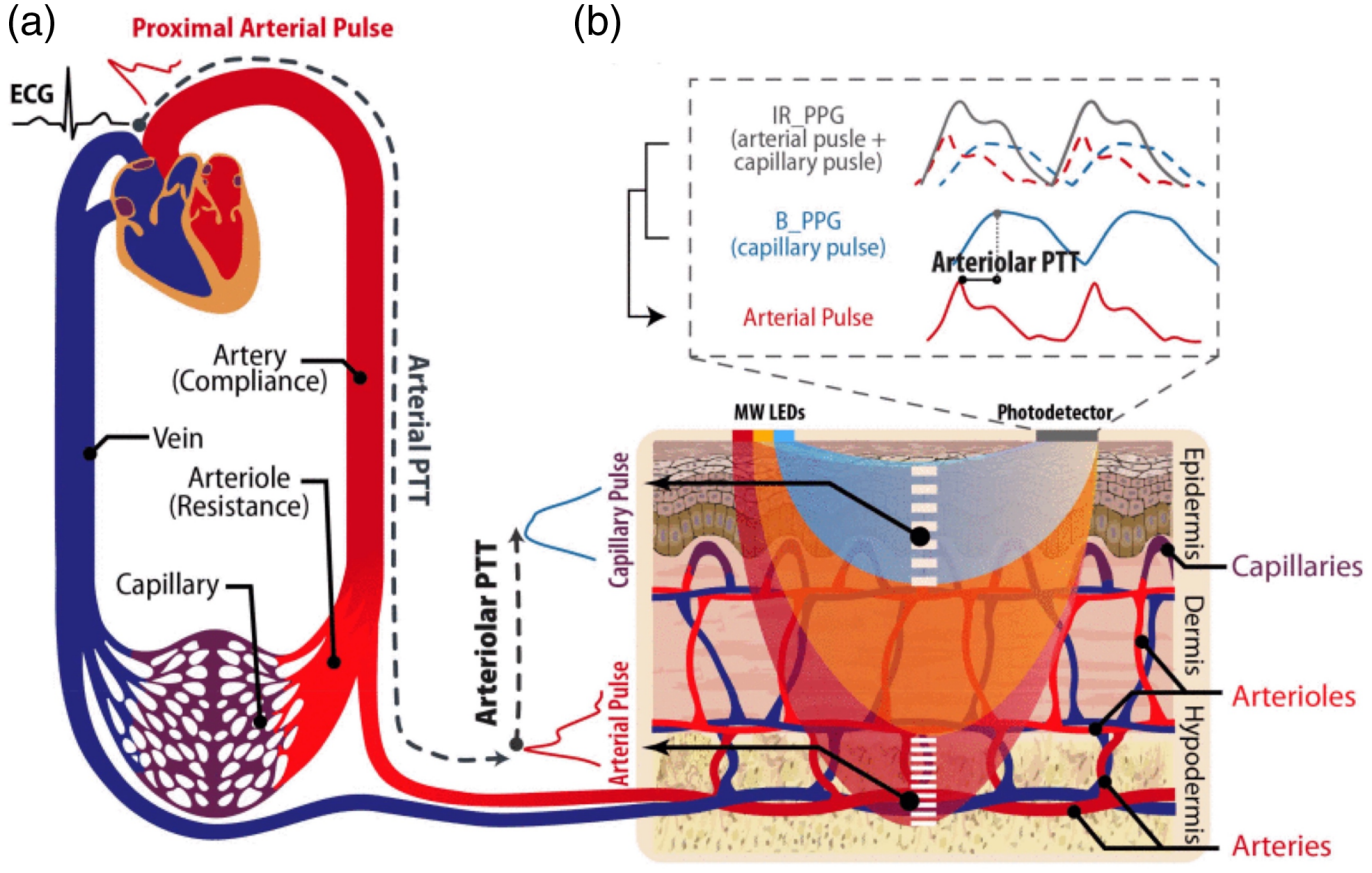
MWPPG measurement of vascular pulsation. (a) Blood propagates through arteries, arterioles, capillaries, and veins found in the epidermis, dermis, and hypodermis. Different wavelengths penetrate into different layers; thus, MWPPG signals are composed of pulsations from different origins. (b) For this simplified example, the infrared PPG signal is considered made up of the arterial and capillary pulsation, whereas the blue PPG signal is composed purely of the capillary pulse. MWPPG signals can therefore be used to extract individual arterial, arteriole, and capillary pulsation. Adapted with permission from Ref. 29.

Liu et al. developed a MWPPG method to isolate the arterial PPG signal from other confounding sources of pulsation in the capillaries and arterioles (Fig. 5). Their study developed an analytical model based on the Beer–Lambert law, using the PPG signals from three shorter wavelengths (470, 570, 590 nm) to remove the capillary and arteriole pulsation from the IR signal (940 nm), leaving behind the purely arterial PPG. The PTT between an ECG and the corrected arterial PPG signal was then calculated and input into a linear regression model to predict systolic blood pressure. They measured 20 subjects with an equal mix of healthy individuals and those with cardiovascular disease. Utilizing the isolated arterial PPG achieved a lower mean absolute difference (MAD) between the predicted and reference SBP measurements: 2.9 mmHg, compared with 5.7 mmHg using the uncorrected IR PPG.^28^

In 2018, the same research group extended their previous work using MWPPG to enable continuous BP measurements with compact wearable electronics. Using the methods from their 2016 paper, the arterial and capillary pulsation was extracted from a four-wavelength MWPPG signal, from which the arteriolar PTT was measured as the time difference between pulse wave arrivals in the capillary and arterial layers. They then created a physiological model relating arteriolar PTT and systemic vascular resistance (SVR), finding that the arteriolar PTT correlated well with SVR. Exploiting this relationship, the Windkessel model was used to ultimately find SBP and DBP. The results demonstrated that the arteriolar PTT-based model outperformed the previously described arterial/ECG PTT model, with an MAD of 2.2 and 1.4 mmHg for SBP and DBP, respectively.^29^ Both studies highlighted the ability of PPG signals isolated from different skin depths to improve blood pressure prediction, facilitated by the use of multiple wavelengths.

Although physiological models for extracting PPG signals at different tissue layers show promise, the increased computational complexity can be a significant limitation for the development of a cuffless wearable MWPPG-based device. Liu et al.^30^ addressed some of these barriers using principal component analysis to separate out the capillary, arterial, and motion artifact-derived pulsations from captured MWPPG signals. They defined the first principal component of the IR PPG pulse as the arterial pulsation, as well as the capillary pulsation and motion artifacts as the first and second principal components from the green (574 nm) and blue (460 nm) PPG signals. The authors then ignored all signal segments marked by high motion artifacts and measured the phase shift between the isolated arterial and capillary pulsation. This phase shift was used to find SBP and DBP through the same analytical models as their previous work, substituting PTT with the phase shift. They measured 22 older adult subjects before and after a 2-h rehabilitation exercise class, using auscultatory BP measurements as ground truth. This technique achieved a mean error of 1.44±6.89  mmHg (MAD 5.51 mmHg) and −1.00±6.71  mmHg (MAD 5.57 mmHg) for SBP and DBP, respectively.^30^

Like single-wavelength PPG, MWPPG signals have also been tested with deep learning approaches as an alternative to the physiological models employed by Liu et al. Cui et al introduced a deep learning model to enhance BP prediction using signals from four wavelengths (660, 730, 850, and 940 nm). The PPG signals from each wavelength were transformed into 2D images using the continuous wavelet transform with the complex Gaussian wavelet basis. These RGB images were then stacked to create a 12-channel input into a convolutional neural network-based bi-directional long short term memory model. The model was tested on 162 volunteers, using 5 s intervals of PPG data and an Omron cuff measurement as the ground truth. Using only a single-wavelength input, the 940 nm signal performed the best, with a mean error of 0.9±7.66 and −0.32±4.07  mmHg for SBP and DBP prediction, respectively. Using all four wavelengths, however, improved SBP and DBP prediction accuracy, resulting in mean errors of 0.22±5.28 and 0.71±2.43  mmHg, respectively. These results demonstrate the potential benefits of using multiple wavelengths to enhance deep learning prediction accuracy for BP estimation.^31^

One challenge for MWPPG techniques is the increased complexity of the hardware implementations, which require multiwavelength sources and/or detection strategies. One example of a compact yet wavelength-rich strategy comes from Chang et al., who proposed a small, low-cost on-chip spectrometer to detect 15 different wavelengths sourced from three broad-spectrum LEDs.^32^ The sensor utilizes plasmonic filters integrated into a photodetector that can synchronously detect five wavelengths within the 505 to 525, 620 to 640, and 930 to 950 nm range. The authors employed cross-correlation techniques to measure PTTs relative to the 505 nm wavelength and averaged over all wavelengths to obtain the average PTT. This PTT was then input into a linear regression model to calculate SBP and DBP. Across 10 subject measurements, the correlation of a ground truth arm-cuff BP measurement with the average PTT was found to be r=0.79 and r=0.78 for SBP and DBP, respectively.^32^

The reviewed studies highlighted the advantages of using multiple wavelengths in PPG measurements for blood pressure estimation. Different wavelengths can penetrate varying depths, thus probing distinct layers of the vasculature. This may help establish a more accurate physiological relationship between the PPG signal and blood pressure while also providing richer input data for machine/deep learning models.^28^^,^^29^^,^^31^^,^^32^ Whether used in physiological models or data-driven approaches, multiwavelength PPG has been shown to improve estimation accuracy. Despite this promise, challenges such as system size, motion artifacts, and signal quality continue to pose limitations.

### 2.5 Speckle Contrast Optical Spectroscopy and Speckle Plethysmography

As previously noted, the PPG signal represents oscillations in the blood volume fraction within the measurement volume, which are caused by the expansion and contraction of arteries and arterioles during the cardiac cycle. It has long been known that the addition of blood flow information to these blood volume changes helps to better characterize the cardiovascular state, i.e., the general status of the cardiovascular system, including BP, HR, vascular compliance, resistance, and tone.^66^ Until recently, high-speed, noninvasive methods for measuring blood flow with the temporal resolution needed to capture detailed waveforms throughout the cardiac cycle were limited. Ghijsen et al.^67^ developed what they termed “wearable speckle plethysmography (SPG),” which uses a laser and a CMOS camera in transmission mode on the finger to measure speckle pattern fluctuations related to blood flow. This technique, also known as speckle contrast optical spectroscopy (SCOS), captures the dynamic interference patterns, or “speckle patterns,” arising from the scattering of laser light on tissue. Such scattering is directly influenced by blood flow.^67^

**Fig. 6:**
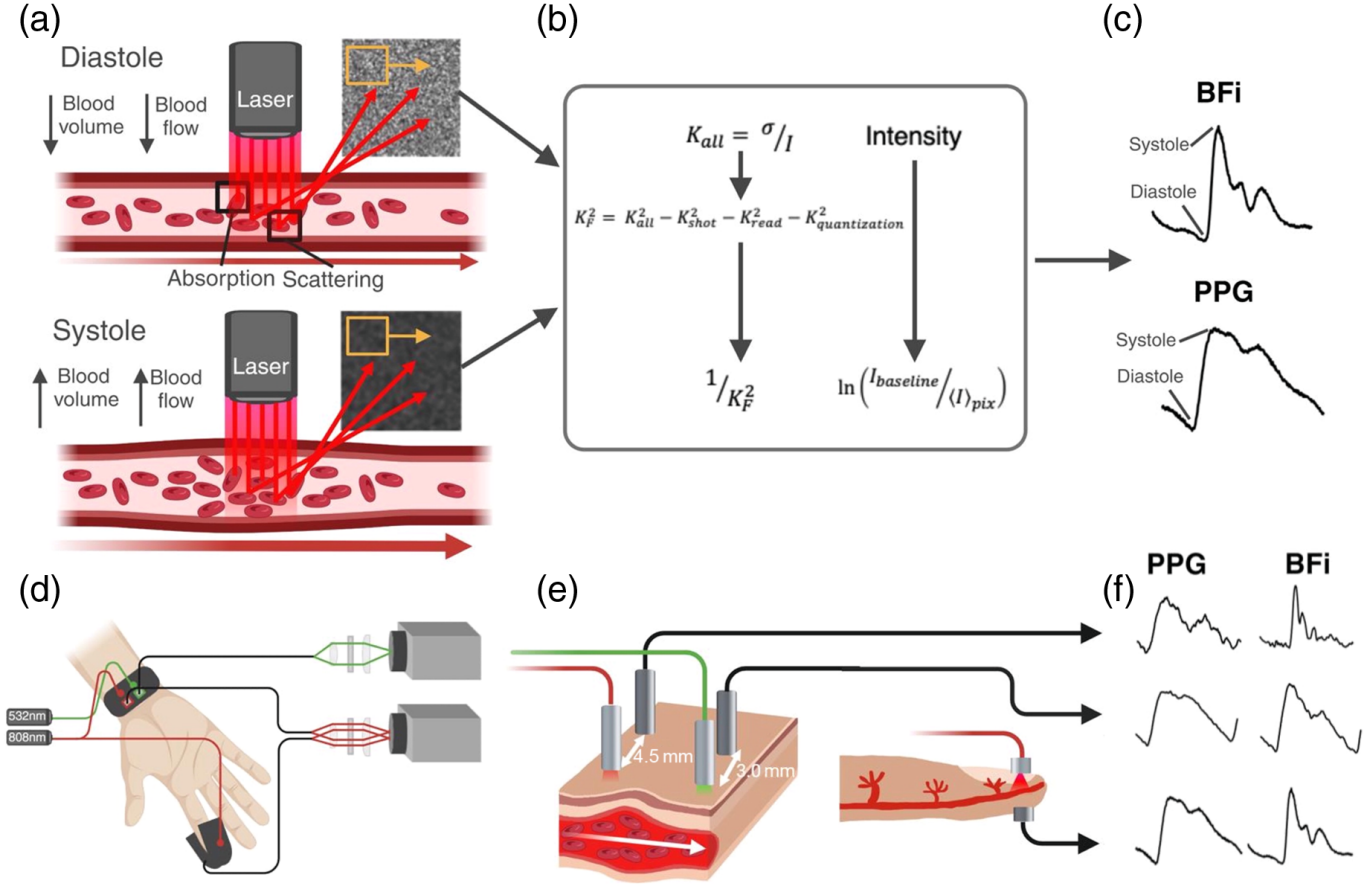
(a) Principle of SCOS measurement. As blood flow and volume increase during systole, speckle contrast and intensity decrease. (b) Data processing pipeline. (c) Representative BFi and PPG waveforms during one cardiac pulse. (d) Measurement set up. (e) 532 and 808 nm reflective measurements are acquired at the wrist. An 808 nm transmission measurement is acquired at the finger. (f) PPG and BFi waveforms at each measurement location. Adapted with permission from Ref. 33 © Optica Publishing Group.

The rate of speckle fluctuations can be quantified by measuring the blurriness of the speckle pattern, termed the speckle contrast (K). K is calculated as the spatial standard deviation of the speckle image divided by its mean intensity. During diastole, the rate of speckle fluctuations is lower due to decreased blood flow, so the blurriness within a given exposure time is decreased and K is increased. Conversely, during systole, blood flow is increased, resulting in increased speckle fluctuations, increased blurriness of the speckle pattern, and decreased speckle contrast [Fig. 6(a)]. Sources of variance unrelated to blood flow changes are subtracted from the speckle contrast, and the relative blood flow index is calculated as $\frac{1}{K_{F}^{2}}$ [Fig. 6(b)]. By acquiring speckle images at a sufficiently high sampling rate, SCOS reveals the pulsatile blood flow index (BFi) waveform.^68^ From these speckle images, the mean intensity can also be measured over time, allowing simultaneous calculation of the PPG signal. The blood flow (BFi) and volume (PPG) signals originate from different physiological sources and thus exhibit different morphology and timing characteristics [Figs. 6(c) and 6(f)].

Ghijsen et al.^67^ explored how the BFi waveforms are related to cardiovascular state. Using a 785 nm laser diode and a CMOS detector, they acquired BFi and PPG waveforms from 16 subjects in transmission mode on the finger.^67^ Subjects underwent cold pressor, exercise, and cuff occlusion tests. They found that the BFi waveforms had higher SNR compared with PPG, particularly during the cold pressor test when perfusion was reduced. This was also observed in a separate study by Herranz Olazabal et al.^69^ Ghijsen et al. also identified consistent time delays between the BFi and PPG systolic peaks; the BFi systolic peaks always preceded the PPG peaks. They hypothesized that the magnitudes of these time delays were related to vascular compliance and were strongly correlated with subject age (vascular stiffness commonly increases with age^70^). They further investigated harmonic features of the BFi waveforms and found that the ratio of the first harmonic to the third harmonic was also correlated with age. Older subjects had less harmonic content in the BFi waveforms, and they hypothesized that this was due to a lower impedance mismatch at vessel bifurcations. In all, this work suggested that the BFi waveforms and their interplay with simultaneously acquired PPG waveforms give a unique window into cardiovascular dynamics at the periphery.^67^ Additional work by Dunn et al. investigated whether BFi waveforms could be used to measure heart rate variability (HRV). They found that the BFi waveforms provided an improved SNR and robustness to motion and temperature compared with PPG. BFi waveforms were obtained from a commercial system, Flowmet, at 785 nm in transmission mode on the finger during rest and an orthostatic exercise. Ground truth HRV was estimated from electrocardiograms. BFi estimated HRV more accurately than PPG across all conditions.^71^

These efforts encouraged investigation into whether BFi waveforms could be used to estimate BP. Garrett et al. were the first to investigate if features extracted from the BFi waveforms were correlated with BP. BFi and PPG waveforms were extracted from 13 subjects at 808 and 532 nm on the wrist and finger in reflective and transmission mode, respectively [Figs. 6(d) and 6(e)]. They compared correlations between BFi, PPG, and joint BFi + PPG features with BP and found that BFi features or features combining information from BFi and PPG waveforms were more strongly correlated with BP than PPG features alone, suggesting that the addition of BFi information could improve BP estimation.^61^ In a second study, they used features from BFi and PPG waveforms on the finger and wrist to predict BP in a cohort of 30 subjects.^33^ Subjects performed a leg press exercise to induce BP changes. Forty features were extracted from the PPG and BFi pulse waveforms. To estimate BP, the eXtreme Gradient Boosting (XGBoost) model was used. Ground truth BP was measured from the Finapres, a volume clamp device that provides continuous blood pressure waveforms. Subject-specific models trained using both BFi and PPG features were compared with models trained using only PPG features. The BFi + PPG model predicted SBP with an MAE of 2.26 mmHg, a 31% improvement in MAE compared with the PPG-only model. To more rigorously evaluate the performance of BP estimates, 20 subjects were remeasured several weeks after the first measurement session. As with the original 30 subjects, both a BFi + PPG model and a PPG-only model were used to estimate BP. However, in this case, the models were trained on the subject’s first measurement session and used to predict BP for the second measurement. Although errors increased, the BFi + PPG model still significantly outperformed the PPG model. The SBP MAE for the BFi + PPG model was 9.85 mmHg, a 19% improvement compared with the PPG-only model. In summary, additional BFi information from SCOS reduced BP errors by 19% to 30% compared with PPG-only, suggesting that SCOS may offer an improved method for cuffless BP estimation compared with PPG-only methods.^33^

The studies described above collected SCOS measurements in contact mode; however, it is also possible to obtain remote SCOS measurements (source and detector not in contact with tissue). Herranz Olazábal et al.^69^ compared the morphological similarity of remote BFi and contact finger transmission PPG signals at 639 nm to finger arterial pressure (fiAP) waveforms from the Finapres in eight subjects. The study computed the MAD between PPG/BFi waveforms and fiAP signals and found that BFi exhibited significantly lower MAD than PPG, suggesting that the BFi waveform is more similar to fiAP. In addition, pulse arrival time (PAT) was assessed relative to ECG, showing that PAT from PPG correlated more strongly with fiAP-derived PAT than BFi-derived PAT.^72^ Ellington et al.^34^ also measured PATs with SCOS and went one step further to estimate BP from the PATs. Eight subjects were measured with SCOS during rest and exercise, and PATs were calculated from the ECG R-peak to the BFi and PPG peaks, respectively. They tested two machine learning models: support vector regression and decision tree regression. Leave-one-subject-out was employed to evaluate errors. Two features were input to the model; the PAT and a calibration BP point for the test subject. When only the PATs were used to train the model, BP errors were high, particularly during exercise (SBP error 16.1 to 20 mmHg). When the calibration point was introduced, errors were reduced to between 2.2 mmHg during light exercise and 19.3 mmHg during heavy exercise. They found that PATs calculated from BFi peaks performed similarly or better than models trained on PATs from PPG peaks, suggesting that PATs calculated from BFi waveforms could provide increased accuracy for BP estimation.

Overall, these studies collectively suggest that BFi information obtained from SCOS contains useful information related to the cardiovascular state that could be used to improve cuffless BP estimations in conjunction with PPG. Broadly, this aligns with accepted knowledge that the relationships between blood flow and volume are related to the cardiovascular state, including blood pressure.^15^^,^^66^ It has been hypothesized that the relationships between BFi and PPG waveforms obtained by SCOS may be closely related to vascular tone, compliance, and resistance in the periphery.^61^^,^^67^^,^^73^ The nature of SCOS enables simultaneous and inherently colocalized measurement of BFi and PPG waveforms. Furthermore, there is growing evidence that BFi offers increased SNR compared with PPG across a wide range of measurement conditions and skin tones.^33^ On the other hand, compared with PPG, it is a more complex measurement, requiring imaging sensors, not just photodiodes, and long-coherence laser sources. Nonetheless, SCOS is a promising new technique for optical cuffless blood pressure measurements. In comparison with the PPG waveform, BFi waveforms are relatively unexplored and may contain as yet unknown features correlated with BP. There has been limited investigation into the effect of wavelength and source-detector separation on BFi waveforms. Such work may further elucidate the relationships between BFi, PPG, and the cardiovascular state.

## 3 BP Estimation Standards

**Fig. 7:**
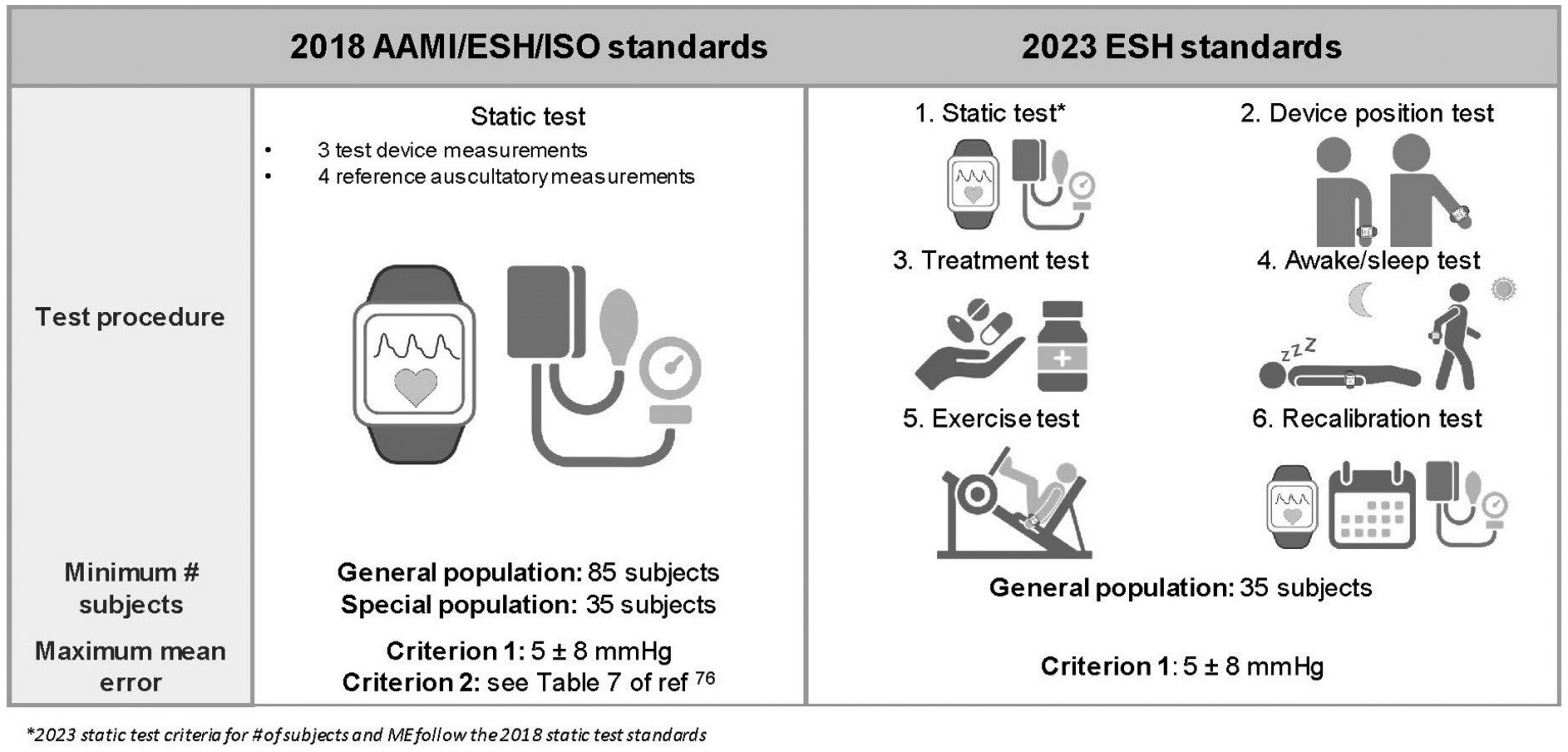
Published standards for new cuffless BP devices. This figure was generated with BioRender.

The studies discussed above have quantified BP errors by comparing to either A-line BP from large databases, single time point cuff measurements, or noninvasive continuous BP measurements from tonometry or volume clamp measurements (e.g., Finapres). The number of subjects varies widely. The types of errors reported also vary, ranging from mean error, root mean square error, mean absolute error, and correlation between ground truth and predicted BP. To address these discrepancies, the Association for the Advancement of Medical Instrumentation, European Society of Hypertension, and the International Organization for Standardization (AAMI/ESH/ISO) collectively published guidelines in 2018 for evaluating the accuracy of new BP devices (Fig. 7).^75^ The guidelines state that at least 85 subjects must be measured three times with the device being tested and four times with an auscultatory cuff BP measurement as the ground truth. There are two criteria for accuracy. Criterion one specifies that, across all BP measurements, the mean error should be less than 5±8  mmHg to be considered accurate. Criterion two specifies that the standard deviation of 85 averaged BP errors (one error per subject) must be within a threshold specified in Table 7 of the guidelines.^76^ In practice, criterion one has been widely used in research studies to contextualize BP errors. As mentioned previously, however, many studies do not meet other criteria laid out in the guidelines even if their errors are low; specifically, insufficient numbers of subjects were enrolled, and the suggested BP method for ground truth was not always used. In many cases, only static tests (measurements at baseline) were used, which is acceptable for evaluating new cuff-based BP measurement devices but not sufficient for a wearable cuffless BP device claiming to track BP changes throughout a user’s daily life.

To address specific challenges and issues related to wearable BP devices, the ESH published additional guidelines for cuffless devices in 2023. The motivation behind these guidelines is that cuffless devices using a calibration point can easily meet the 2018 AAMI/ESH/ISO guidelines, which only require BP measurements at baseline during one sitting. However, meeting these guidelines does not show that the device can accurately track BP over time, which is the typical goal of a cuffless BP device. As such, the updated guidelines require six tests for cuffless devices, rather than a single measurement at rest.^74^ The six tests are shown in Fig. 7. Preliminary studies investigating the accuracy of new optical cuffless BP devices may find it difficult to implement all six of these tests, particularly the treatment test and the awake/sleep test, given the demanding human subject protocols and the requirement for at least a semi-wearable device. BP perturbations and longitudinal measurements are relatively straightforward, requiring only an exercise component and repeated measurements, and should be implemented in studies evaluating such devices.

## 4 Commercially Available Optical BP Monitoring Devices

Table 2 shows currently approved cuffless BP devices. To our knowledge, there are only two FDA-cleared devices that use only optical measurements, which are the BioBeat device (FDA K numbers K222010, K190792, and K241066) and Aktiia’s G0 Blood Pressure Monitoring System (FDA K number K250415). The BioBeat device relies on pulse wave analysis (PWA) of PPG signals on the dorsal side of the wrist. The sensor has four LEDs at the red and NIR wavelengths that are also used to measure ${\mathrm{SpO}}_{2}$. The device uses pulse wave transit times (PWTT), which are likely calculated from PPG signals at different wavelengths, similar to Liu et al.,^29^ or perhaps different source detector separations (specific details are not specified in the associated FDA K documents or available manufacturer documentation). BioBeat received clearance based on the 2013 ISO standard for automated noninvasive BP cuffs, which is similar to the AAMI/ESH/ISO 2018 guidelines, and they cite an accuracy of ±5  mmHg.^77^ The device requires an initial calibration using three cuff BP measurements. The G0 blood pressure monitoring system similarly relies on PWA of PPG signals at the wrist, though utilizing a green LED. The device received clearance based on the 2018 ISO standard for noninvasive sphygmomanometers. As mentioned previously, the guidelines used to clear both the BioBeat and Aktiia G0 devices may not be sufficient to assess cuffless BP device accuracy under real-world use cases.

**Table 2:** Commercially available optical BP monitoring devices.

Device manufacturer	Device type	Method	Regulatory approval
BioBeat	Chest/hand wearable	PWTT (distal vessel PPG)^a^	FDA cleared
Aktiia	Wristband	PWA (distal vessel PPG)	FDA cleared
Sotera	Chest/hand wearable	PWA (thumb PPG) + PAT (ECG)	FDA cleared
Samsung	Smartwatch	PWA (distal vessel PPG)	S. Korea approved
Sensifree	Finger clip	PWA (distal vessel PPG)	CE marked
Biospectal	Fingertip measurement with a smartphone	PWA (distal vessel PPG)	CE marked
Sky labs	Finger ring	PWA (distal vessel PPG)	S. Korea approved

BP, blood pressure; PWTT, pulse wave transit times; PPG, photoplethysmography; PWA, pulse wave analysis; PAT, pulse arrival time; ECG: electrocardiogram; FDA, Food and Drug Administration; CE, European Conformity.

a The BioBeat device uses PWTT from multiwavelength and/or multidistance PPG measurements.

In a recent study conducted by BioBeat, 30 subjects were measured with both the BioBeat wrist device and an ABPM. Low errors within the AAMI guidelines were reported when comparing the average SBP and DBP obtained over the 24 h. The 95% limit of agreement for 24H-averaged measurements was [−4.1,1.8] and [−4.2,1.9]  mmHg for DBP and SBP, respectively. However, when comparing errors for individual cuff measurements over 24 h rather than the average, errors were significantly higher.^78^ In this case, the 95% limit of agreement was [−17.38,15.12] and [−15.02,12.83]  mmHg for DBP and SBP, respectively. Similarly, Tan et al.^79^ showed that the Aktiia device could not measure nighttime dipping accurately, which is an important metric from ABPM. The Atkiia device reported an average nighttime dip in SBP and DBP of −5.1 and −3.8  mmHg, respectively. This is substantially lower than the average SBP/DBP nighttime dip as measured by the ABPM, reported to be −18.0 and −13.8  mmHg.

Table 2 shows other commercially available cuffless BP devices, many of which have conflicting reports on their accuracy. For example, Falter et al.^80^ showed that the Samsung wrist device had a systematic bias toward the calibration measurement. The OptiBP app requires users to place their finger on the smartphone to acquire waveforms, which cannot provide continuous BP measurements. Sky Labs developed a finger ring for BP monitoring, which has been validated against A-line BP measurements but not throughout the subject’s daily lives.^81^ Perhaps most importantly, the Microsoft Research Aurora project investigated the accuracy of wearable BP devices on a large scale. They measured 1125 subjects with wearable BP devices that used PWA, PAT from ECG and PPG, and tonometry to estimate BP. They compared their BP estimation results using these devices to a model trained only on an initial calibration and the time of day. They found that models trained with tonometry or PPG information contributed little value to measuring resting or ambulatory BP, casting doubt on the accuracy of currently available commercial devices.^82^ Independent, larger-scale studies evaluating these devices are needed to fully assess accuracy.

## 5 Discussion

Here, we have reviewed current optical techniques for cuffless blood pressure estimation, including finger PPG, wrist PPG, imaging PPG, multiwavelength PPG, and speckle contrast optical spectroscopy (SCOS)/speckle plethysmography (SPG). Across these various modalities, similar data processing and BP prediction strategies were employed. Physiological models, such as the Windkessel model, have been utilized with PPG as a basis for BP prediction. However, the majority of studies rely on machine learning or deep learning approaches. Feature-based machine learning is the most commonly used method for extracting BP from PPG, where relevant features from the optical signal are identified and fed into an algorithm. Features derived from the second derivative of the PPG signal or its frequency domain representation are also often incorporated.^83^ A broad range of machine learning and deep learning techniques have been applied for BP estimation.^16^^,^^84^^–^^89^ However, one challenge in feature-based machine learning is the extraction of relevant PPG features from large datasets. PPG signal morphology varies between individuals, and noisy or low-quality signals can hinder reliable feature extraction. Techniques such as noise reduction, automatic outlier removal, and signal filtering can enhance feature extraction. Instead of manually selecting features from the PPG signal for input into a classification algorithm, some approaches use the whole PPG signal as a feature vector input into a deep learning algorithm.^18^^,^^19^^,^^87^^,^^88^^,^^90^^,^^91^ This method can improve BP prediction, particularly in cases of noisy or atypical PPG signals. However, deep learning models are a “black box,” making it difficult to interpret which elements of the input signal are contributing to BP predictions.

The majority of research effort has gone toward predicting BP from the finger PPG signal.^36^ The finger PPG signal has several advantages; first, it tends to have a higher SNR than the PPG signal at other locations, and second, there are large databases with concurrent finger PPG and A-line BP measurements that can be used to build large BP prediction models, including the MIMIC database and the University of Queensland dataset.^20^^,^^33^^,^^61^^,^^92^ However, models built on these datasets are acquired from ICU patients with A-line BP measurements and may not be applicable to healthy populations outside of the clinic. Despite a significant amount of effort toward predicting BP from the finger PPG signal, there is still no widely used finger PPG BP monitor.

On the other hand, the popularity of wrist-worn wearable devices has encouraged increasing research into wrist PPG signals for BP estimation.^20^^–^^22^^,^^37^^,^^93^^–^^95^ There is an ongoing debate about the physiological origin of these reflective PPG signals, as well as the ideal measurement location on the wrist.^48^^–^^50^^,^^59^ Wrist PPG signals tend to be noisier than finger PPG signals and more dependent on measurement location. Future research should aim to better understand the ideal wavelengths and locations for acquiring wrist PPG signals, which may enable improved BP estimation.

The COVID-19 pandemic increased motivation for contactless health monitoring, which led to an increase in studies using imaging PPG (iPPG) for BP estimation.^23^^,^^24^^,^^27^^,^^54^ iPPG does not require contact between optical probes and the skin and can be obtained with room light; however, it is more sensitive to motion and tends to produce noisier PPG signals.^62^ iPPG is typically acquired from the face and cheek regions, which makes it difficult to provide wearable, continuous BP monitoring. Rather, it is more likely to be applied for single snapshots of BP or continuous monitoring in a clinical bedside scenario.

Across different measurement locations, multiwavelength PPG has been of increasing interest as a method to better PPG-based BP prediction. Multiwavelength PPG (MWPPG) uses multiple light sources at different wavelengths to probe the PPG signal at different tissue volumes.^28^^–^^31^ The ability of different wavelengths to penetrate various vascular layers may help establish a more precise physiological relationship between PPG and BP while also enriching the data used for machine learning predictions, which may improve BP estimation accuracy.

Besides PPG-based BP prediction, speckle contrast optical spectroscopy/speckle plethysmography (SCOS/SPG) measurements have also demonstrated potential for use in BP estimation. SCOS/SPG uses a laser and camera to measure both blood flow (BFi) and volume (PPG) waveforms simultaneously.^33^^,^^34^^,^^61^^,^^67^^,^^69^^,^^71^^,^^72^^,^^96^ The BFi waveforms have been shown to have higher SNR than PPG signals across different levels of perfusion.^33^^,^^67^^,^^69^ Features combining information between BFi and PPG, and BFi features alone, are strongly correlated with age and blood pressure.^61^ Preliminary attempts to estimate BP from BFi and PPG signals combined showed that adding BFi information improved the BP estimation by up to 31% compared with a model using only PPG.^33^ This research collectively suggests that SCOS measurements contain abundant, yet relatively unexplored, information related to cardiovascular health. One disadvantage of SCOS is that it requires lasers and imaging sensors, which is more complex than what is required to obtain a typical PPG signal. More work is needed to fully understand the relationship between the BFi, PPG, and BP signals.

Despite many efforts to develop accurate, cuffless optical BP monitoring, only two optical devices (BioBeat and Aktiia) have obtained FDA clearance to our knowledge.^97^ Although preliminary studies using the devices in comparison with ABPM are promising, larger scale studies are needed to fully validate these devices. Particularly, the large-scale Microsoft Aurora Research Project casts some doubt on the ability of current commercial devices to accurately measure BP. On more than 1000 patients, they found that utilizing pulse wave analysis-based ML models from PPG or tonometry signals (similar to commercially available devices) contributed no value to BP prediction over models based solely on an initial calibration and time of day.^82^ This suggests that more research is needed to improve optical BP estimation. Techniques such as iPPG, MWPPG, and SCOS are relatively new and could provide avenues for improved BP estimation. More research exploring the physiological origins of these signals and the impact of different optical configurations on BP estimation may improve accuracy. Combining information from different optical techniques may improve BP estimation.

Future studies evaluating new wearable BP devices should make every effort to follow the updated 2023 ESH guidelines for new cuffless devices. These updated guidelines require six validation tests, rather than measurements only at baseline. As discussed previously, early studies testing new devices may find it difficult to implement all six tests, but adding BP perturbations or longitudinal measurements is typically not difficult to incorporate. New devices should strive to measure BP at the rate of ABPM (every 30 to 60 min) or faster. Due to the lack of continuous BP monitors available, there is no clinical evidence that measuring BP at a higher sampling rate is effective for hypertension diagnosis. However, it is reasonable to expect that a wearable, noninvasive device capable of measuring short-time scale changes in BP would have clinical value.

## 6 Conclusion

Although significant progress has been made in optical, cuffless BP monitoring, challenges remain in achieving widespread clinical adoption. The integration of novel techniques such as iPPG, MWPPG, and SCOS presents promising opportunities for improving BP estimation accuracy. However, further research is needed to refine signal processing techniques, enhance model generalizability, and validate these approaches in large-scale clinical studies. As new wearable BP devices emerge, adherence to standardized validation protocols, such as the updated 2023 ESH guidelines, will be crucial to ensuring reliability and regulatory approval. Further research into the physiological origins of the signals and the impact of illumination wavelength and source detector separation may lead to improvements in optical configurations for wearable BP devices. Combining different modalities and increasing the depth of information obtained from the optical signals could also improve the accuracy of BP estimation. Continued effort toward developing and improving optical BP monitoring may eventually realize a major goal of the biomedical community: continuous, cuffless blood pressure monitoring.

## Supplementary Material

10.1117/1.BIOS.3.1.010901.s01

## Data Availability

Data sharing is not applicable to this article, as no new data were created or analyzed.
